# Inoculation

**Published:** 1800-07

**Authors:** 


### INOCULATION.

At a time when the Vaccine Inoculation is nearly eſtabliſhed in this iſland, and when it has made a conſiderable progreſs in Ireland and on the continent, it may gratify the curioſity of many of our readers to be informed what kind of reception the Inoculation of the Small-Pox experienced on its firſt introduction into this kingdom. With this view we preſent them with the following account. In the year 1746, a ſmall pamphlet was publiſhed, intitled,

"*Several Cases in Physic; and one in particular, giving an Account of a Person who was inoculated for the Small-Pox, and had the Small-Pox upon the Inoculation, and yet had it again; together with two or three other very remarkable Small-Pox Cases, &c.* By Pierce Dod, M.D. Fellow of the College of Phyſicians, and Phyſician to St. Bartholomew's Hoſpital."

The particular Caſe referred to in the above title-page is given as follows, at page 12 of the pamphlet.

"I did not think to trouble you with any thing more at preſent, but another Small-pox caſe being lately come to my hands, I was unwilling to loſe the opportunity of communicating it.

"It

"It is the cafe of a little boy, fon to Mr. Richards, member of parliament for Bridport in Dorfetfhire. He was inoculated about three years ago, at the age of about three, and had the Small-pox come out upon him to the number of fifty or fixty, and they maturated and fcabbed, and went off in the fame manner that that fort of pock, which was a regular and diftinct one, generally does: But about two years afterwards, *i. e.* about a year ago, he had the Small-pox again, and there came out between two and three hundred, which maturated and fcabbed likewife, and went through the fame ftages, and in the fame manner that the former did, only he was worfe before the eruption; but when that was completed, had no manner of diforder, except too much of an appetite, as is ufual, every now and then, in and after the Small-pox.

"This is authenticated to me by a letter which I have from a learned and experienced phyfician in thofe parts, Dr. Broorepp, who is likewife grandfather to the child, and attended him upon both occafions; and, whatever may be faid in evafion of other cafes, in which perfons that have been inoculated have had the Small-pox afterwards, as,—that the inoculation did not take place, there was no eruption, at leaft no variolous one, and the like,—is certainly an inftance that *all* is not to be depended upon that is given out in favour of, or is expected from Inoculation; and that it is, by no means, an effectual fecurity from having the Small-pox again.

"But, as it has been obferved long fince by the inimitable author of HUDIBRAS,

> "Surely the pleafure is as great
> "In being cheated, as to cheat."

or elfe it is impoffible that Inoculation fhould triumph fo much as it is faid that it does over all its oppofers."

This curious pamphlet was anfwered by Kirkpatrick, in the fame year, with great wit and feverity, under the following title:

"*A Letter to the real and genuine Pierce Dod, M. D. actual Physician to St. Bartholomew's Hospital; plainly exposing the low Absurdity or Malice of a late spurious Pamphlet, falsely ascribed to that learned Physician: with a full* ANSWER *to the* MISTAKEN *Case of a natural Small-pox, after taking it by Inoculation.*"

Thefe two interefting pamphlets are become very fcarce; though the latter was afterwards reprinted with the author's real name, and an apology for the feverity of fome of his animadverfions, which he underftood had diminifhed Dr. Dod's reputation and practice.

